# Impact of the National Essential Public Health Service Package on Blood Pressure Control in Chinese People With Hypertension: Retrospective Population-Based Longitudinal Study

**DOI:** 10.2196/65783

**Published:** 2025-02-06

**Authors:** Feiran Wei, You Ge, Han Li, Yuan Liu

**Affiliations:** 1 School of Public Health, Southeast University, Nanjing, China; 2 Department of Infectious Disease, The Second Hospital of Nanjing, Nanjing, China; 3 Institute for Chronic Disease Management, Jining No. 1 People’s Hospital, 0802 Huoju, Jining, 272000, China, +86 19853782628

**Keywords:** hypertension, primary care, public health, blood pressure control, cardiovascular disease

## Abstract

**Background:**

The National Essential Public Health Service Package (NEPHSP) was launched in 2009 to tackle poor blood pressure control in Chinese people with hypertension; however, it’s effect is still unclear.

**Objective:**

In a retrospective population-based longitudinal study, we aimed to evaluate effect of the NEPHSP on blood pressure control.

**Methods:**

A total of 516,777 patients registered in the NEPHSP were included. The blood pressure control data were assessed based on the Residence Health Record System dataset. We longitudinally evaluated the effects of the NEPHSP on blood pressure control by analyzing changes in blood pressure at quarterly follow-ups. Both the degree and trend of the blood pressure changes were analyzed. We conducted stratified analysis to explore effects of the NEPHSP on blood pressure control among subgroups of participants with specific characteristics.

**Results:**

The mean baseline systolic blood pressure (SBP) and diastolic blood pressure (DBP) were 147.12 (SD 19.88) mm Hg and 85.11 (SD 11.79) mm Hg, respectively. The control rates of baseline SBP and DBP were 39.79% (205,630/516,777) and 69.21% (357,685/516,777). Compared to baseline, the mean SBP decreased in each quarter by 5.06 mm Hg (95% CI −5.11 to −5.00; *P*<.001), 6.69 mm Hg (95% CI; −6.74 to −6.63; *P*<.001), 10.30 mm Hg (95% CI −10.34 to −10.23; *P*<.001), and 6.63 mm Hg (95% CI −6.68 to −6.57; *P*<.001). The SBP control rates increased in each quarter to 53.12% (274,493/516,777; β coefficient=0.60, 95% CI 0.59-0.61; *P*<.001), 56.61% (292,537/516,777; β coefficient=0.76, 95% CI 0.75-0.77; *P*<.001), 63.4% (327,648/516,777; β coefficient=1.08, 95% CI 1.07-1.09; *P*<.001), and 55.09% (284,711/516,777; β coefficient=0.69, 95% CI 0.68-0.70; *P*<.001). Compared to baseline, the mean DBP decreased in each quarter by 1.75 mm Hg (95% CI −1.79 to −1.72; *P*<.001), 2.64 mm Hg (95% CI −2.68 to −2.61; *P*<.001), 4.20 mm Hg (95% CI −4.23 to −4.16; *P*<.001), and 2.64 mm Hg (95% CI −2.68 to −2.61; *P*<.001). DBP control rates increased in each quarter to 78.11% (403,641/516,777; β coefficient=0.52, 95% CI 0.51-0.53; *P*<.001), 80.32% (415,062/516,777; β coefficient=0.67, 95% CI 0.66-0.68; *P*<.001), 83.17% (429,829/516,777; β coefficient=0.89, 95% CI 0.88-0.90; *P*<.001), and 79.47% (410,662/516,777; β coefficient=0.61, 95% CI 0.60-0.62; *P*<.001). The older age group had a larger decrease in their mean SBP (β coefficient=0.87, 95% CI 0.85-0.90; *P*<.001) and a larger increase in SBP control rates (β coefficient=0.054, 95% CI 0.051-0.058; *P*<.001). The participants with cardiovascular disease (CVD) had a smaller decrease in their mean SBP (β coefficient=−0.38, 95% CI −0.41 to −0.35; *P*<.001) and smaller increase in SBP control rates (β coefficient=−0.041, 95% CI −0.045 to −0.037; *P*<.001) compared to the blood pressure of participants without CVD.

**Conclusions:**

The NEPHSP was effective in improving blood pressure control of Chinese people with hypertension. Blood pressure control of older individuals and those with CVD need to be intensified.

## Introduction

The Global Burden of Disease Study reported that cardiovascular diseases (CVDs) are the leading noncommunicable disease contributing to the increase in disease-adjusted life years in China [1-3]. Hypertension, the major modifiable risk factor for CVDs, is prevalent in China [4-12]. According to the data of a recent survey conducted nationwide, the prevalence of hypertension was 54.7% among Chinese adults over 18 years old, and reportedly, merely 60.1% of Chinese adults with hypertension accepted antihypertensive treatments [13]. The low hypertension treatment rate has led to a suboptimal control of blood pressure among Chinese adults with hypertension [13]. Merely 7.2% of Chinese adults with hypertension had their blood pressures under control [13].

To tackle the poor hypertension control and growing burden of CVDs, the Chinese government launched the National Essential Public Health Service Package (NEPHSP) in 2009 [14]. The NEPHSP was a set of public health services which were available for all community-dwelling residents [14-18]. The public health services contain 4 modules, which are screening, monitoring, regular follow-up, and individualized interventions for hypertension control. The 4 modules were provided by primary health care professionals [14-18]. NEPHSP registration has been continuously expanded since it was launched in 2009 [14,18]. A total of 109 million Chinese adults diagnosed with hypertension registered with the NEPHSP from 2009 to 2019 [14]. The government investment increased from 15 to 84 RMB (US $2.05 to US $11.46) per person annually [15,18]. Because of the registration expansion and investment increase for the NEPHSP, the proportion of Chinese adult with hypertension who accepted antihypertensive treatment increased by 59.58% from 2009 to 2019 [14,18].

Although the registration expansion and investment increase for the NEPHSP improved the hypertension treatment rate, the NEPHSP lacks quality-oriented evaluations, which leads to an uncertainty whether the NEPHSP is effective for blood pressure control [14-18]. Furthermore, research demonstrating the effectiveness of the NEPHSP for blood pressure control in Chinese adults with hypertension is lacking [14-18]. To fulfill the research gap, this study aimed to explore effect of the NEPHSP on blood pressures control of Chinese adult with hypertension.

## Methods

### Study Design

This study was designed as longitudinal study to evaluate effect of the NEPHSP intervention on blood pressure control. The longitudinal study observed changes in the health status and clinical indicators of individuals who accepted treatment in a period of time, in order to explore the association between treatment and disease control [19-22]. Longitudinal studies have been commonly used in clinical research to evaluate the effects of treatment [19-22]. In this longitudinal study, we repetitively measured the blood pressure in participants who accepted hypertension care services offered by the NEPHSP in each quarter of 2023. All participants had a recorded blood pressure measurement prior to joining the NEPHSP in 2022. With the baseline outcomes in 2022 as the control, the degree and trend of the blood pressure changes after receiving NEPHSP in 2023 were analyzed longitudinally.

The data in this study were extracted from the Residence Health Record System, which is an electronic health record system built to recording demographic and health information of people registering with the NEPHSP [16,17]. This study used data of the Residence Health Record System built in Jining City, which is a national pilot city of the Chronic Disease Comprehensive Prevention and Control programs and is located in eastern China.

### Study Participants

**Figure 1.**
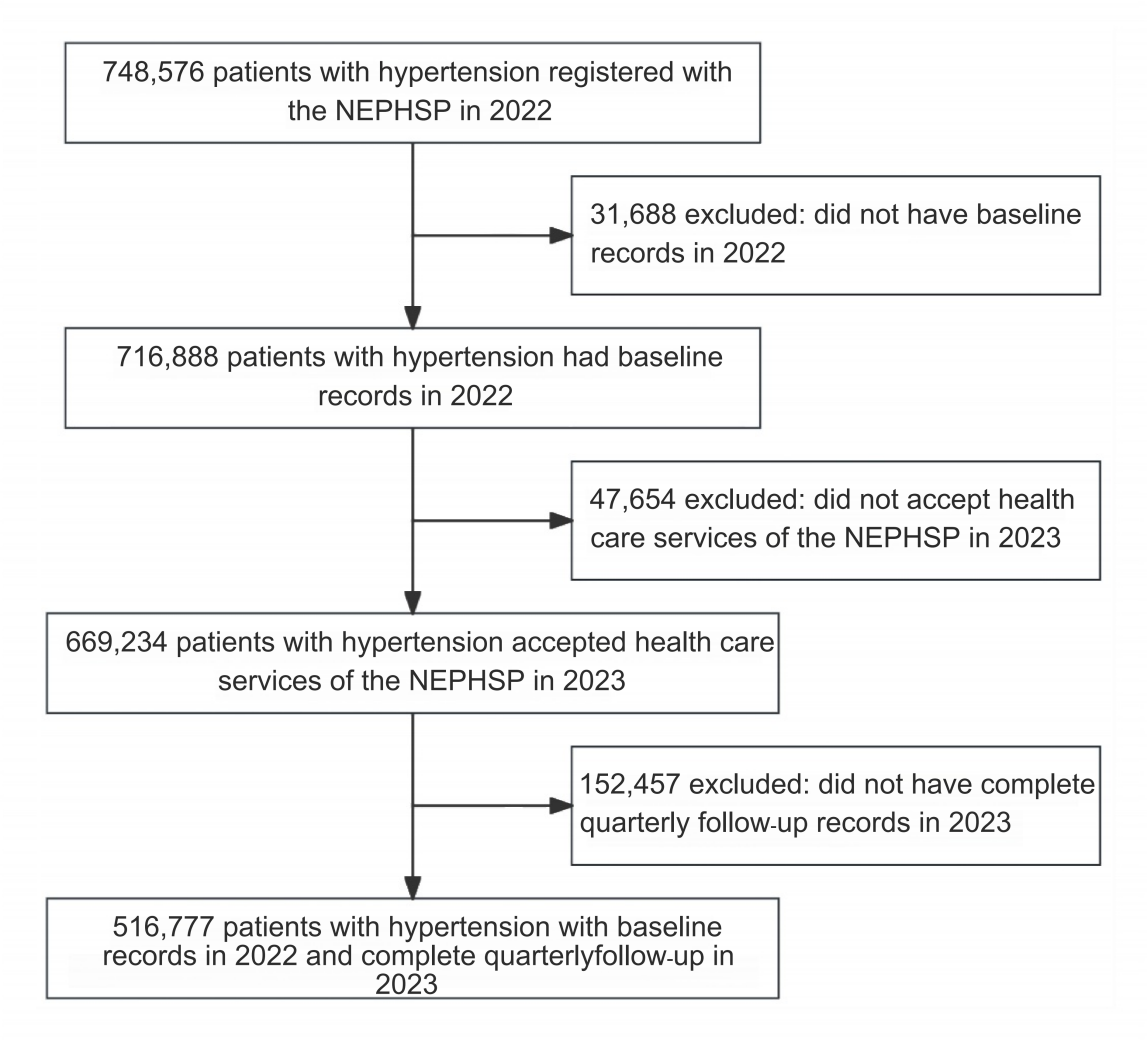
The flowchart of the participant inclusion. NEPHSP: National Essential Public Health Service Package.

We analyzed data from the NEPHSP participants from 1593 primary care institutions in the 9 districts in Jining City. Eligibility criteria were (1) individuals registering with the NEPHSP as a patient with hypertension, (2) individuals who had baseline records of health examinations in 2022, (3) individuals who accepted health care services offered by the NEPHSP in 2023, and (4) individuals who had records for quarterly follow-ups conducted in 2023. The flowchart of participant inclusion is described in Figure 1.

### Intervention

**Figure 2.**
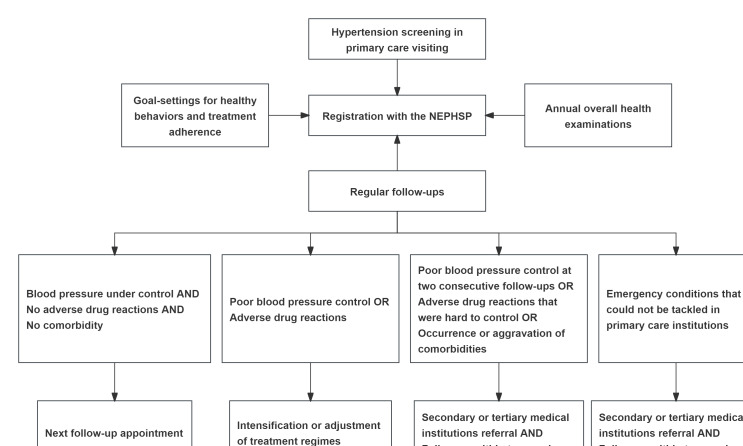
Flowchart of the NEPHSP. NEPHSP: National Essential Public Health Service Package.

The NEPHSP was divided into 4 dimensions: screening, routine follow-ups, individualized interventions, and annual health examinations. The explicit flowchart of the NEPHSP is described in Figure 2.

The screening was conducted at primary care institutions when community residents had an appointment with primary health care professionals. The criteria for a hypertension diagnosis were a systolic blood pressure (SBP) over 140 mm Hg or a diastolic blood pressure (DBP) over 90 mm Hg for 3 separate measurements on different days. The residents were invited to register with the NEPHSP if they were diagnosed with hypertension.

The routine follow-up contained 5 facets: (1) assessing emergency conditions, (2) evaluating hypertensive symptoms, (3) measuring blood pressure, (4) evaluating healthy behaviors, and (5) evaluating medication treatment regimens and compliance. Assessing emergency conditions was the first part of the routine follow-up. The participants would be referred to secondary or tertiary care settings if there were emergency conditions that were hard to tackle in primary care institutions. If no emergency conditions were identified, the remaining parts would be conducted. The regular follow-ups were provided quarterly.

The individualized interventions contained 3 components: medication treatment adjustment, referral channel to superior medical institutions, and healthy behavior changes. According to health conditions that were evaluated in the regular follow-ups, primary care professionals would make decisions on medication treatment adjustments for the participants. If the health issues could not be handled in primary care settings, the primary care professionals would transfer the patient to superior medical institutions. The explicit processes and criteria of medication treatment adjustment and referral were divided into 3 dimensions. First, if blood pressure was under control, there were no adverse drug reactions, and there was not an occurrence of comorbidities or an aggravation of comorbidities, the next follow-up appointments were made for the participants. Second, if blood pressures were uncontrolled or adverse drug reactions were identified, the primary health care professionals would adjust the antihypertensive medication regimens. The conditions of the participants would be re-evaluated within 2 weeks. Third, if the patients had refractory uncontrolled blood pressure, adverse drug reactions that could not be tackled in primary care settings, or an occurrence and aggravation of comorbidities, the primary health care professionals would refer the participants to secondary or tertiary care settings. The conditions of the participants would be re-evaluated within 2 weeks.

Changes in health behavior were available for all participants, and goal-setting was used to formulate their health behaviors. The primary health care professionals developed shared goals with the participants. The targets for changes in health behavior included medication treatment compliance, dietary changes and weight control, control of smoking and alcohol drinking, and exercise engagement. The achievements of the health behavior changes were evaluated at the routine follow-ups. Annual health examinations were available for all patients. The health examinations contained overall physical and bioclinical examinations, as well as lifestyle and medication treatment evaluations.

### Outcome Variables

We evaluated the impact of the NEPHSP on blood pressure control in Chinese patients with hypertension through changes in SBP and DBP and changes in the control rates of SBP and DBP. The control rates of SBP and DBP were the proportion of study participants whose SBP and DBP values were in the normal range, respectively. The normal range of SBP and DBP in the Chinese hypertension guideline is defined as <140 mm Hg and <90 mm Hg, respectively [23].

We used the mean blood pressure values at baseline in 2022 and at 4 quarterly follow-up visits in 2023 to depict trends in blood pressure for the patients before and after receiving the NEPHSP services. The baseline values of SBP and DBP were averaged by the blood pressure measurements of the health examinations in 2022. The mean follow-up values of SBP and DBP were averaged by blood pressure measurements of the routine follow-ups. We extracted the blood pressure data from the Residence Health Record System, which recorded the SBP and DBP values measured in the annual health examinations and the regular follow-ups of the NEPHSP participants.

### Statistical Analysis

Baseline characteristics of the participants were statistically described. The quantitative variables were described as mean and SD, while categorical variables were described as counts and percentage. The generalized estimating equation (GEE) model was used to analyze the repetitively measured blood pressure data that contained a continuous time variable [24]. The GEE model was capable of analyzing numerical and categorical data that were repetitively measured [24]. Both the trend and degree of the blood pressure changes were analyzed. First, the variable of time was set as the continuous variable to analyze the trend of the blood pressure changes in the GEE models. Linear trend tests were conducted to examine whether the changes in the blood pressure values with the continuous time variation were linear. The linear trend test were reflected by *P* for trend [25,26]. If the changes in the blood pressure values for the continuous time variation were linear, we used the coefficients of GEE models to reflect linear trends of blood pressure changes. Second, we also set the variable of time as the categorical variable to analyze the degree of blood pressure changes in the GEE models. The baseline blood pressure values in 2022 were chosen as the reference group. All statistical analyses were carried out by R software version 4.0.4 (R Core Team). The analysis was performed with 2-tailed tests at an α level of .05.

The covariates were first selected based on evidence of previous studies that had proven the factors that could impact blood pressure [27-34]. Subsequently, we also conducted univariate analyses to confirm whether the covariates had an impact on blood pressure. The following participant characteristics were adjusted as covariates: age, sex, BMI, waist circumference, exercise engagement, alcohol drinking and smoking status, fasting plasma glucose, total cholesterol, triglycerides, low density lipoprotein, high density lipoprotein, baseline SBP and DBP values, diagnosis of cardiovascular and renal diseases, and duration since hypertension diagnosis. The results of univariate analyses and corresponding information of each covariate in the GEE models are described in Multimedia Appendix 1 and Multimedia Appendix 2.

### Sensitivity and Stratified Analyses

A series of stratified and sensitivity analyses were conducted to identify whether the changes in blood pressure were different by subgroup. The subgroups were predefined as an older age group (≥65 years old) and younger age group (<65 years old), a male group and female group, a CVD diagnosis group and no CVD diagnosis group, and a baseline controlled SBP group and baseline uncontrolled SBP group. We fit GEE models to assess the changes in blood pressure separately for those subgroups. We then tested the interactive effects between the time variables (continuous time variable) and the subgroup indicators using the total sample, to formally evaluate whether the changes in blood pressure differed between the subgroups.

### Ethical Considerations

The study was conducted in accordance with the Declaration of Helsinki and approved by the Committee on Human Research of the Jining No. 1 People’s Hospital (2023 Ethical Approval No. KYLL-204609‐176). The need for informed consent was waived due to the retrospective nature of this study.

## Results

### Baseline Characteristics

The baseline characteristics are accessible in Table 1. A total of 516,777 participants were involved in this study. The mean age was 68.59 (SD 9.57) years, 58.56% (n=302,109) of the participants were female, and 66.94% (n=345,923) of the participants were over 65 years old. The mean values for baseline SBP and DBP were 157.12 (SD 19.88) mm Hg and 85.11 (SD 11.79) mm Hg, respectively. The mean values of baseline SBP were above the normal criteria [19]. The control rates of baseline SBP and DBP were 39.79% (205,630/516,777) and 69.21% (357,685/516,777), respectively.

**Table 1.** Baseline characteristics.

Characteristics	Values
Age (years), mean (SD)	68.39 (9.57)
Sex, n/N (%)	
Male	214,668/516,777 (41.54)
Female	302,109/516,777 (58.56)
DBP^a^ (mm Hg), mean (SD)	85.11 (11.79)
DBP control rate, n/N (%)	357685/516,777 (69.21)
Cardiovascular disease diagnosis, n/N (%)	124409/516,777 (24.07)
SBP^b^ (mm Hg), mean (SD)	
All participants	147.12 (19.88)
Older participants	149.81 (20.37)
Younger participants	141.67 (17.64)
Participants with cardiovascular disease	149.14 (18.48)
Participants without cardiovascular disease	144.47 (20.02)
SBP control rate, n/N (%)	
All participants	205,630/516,777 (39.79)
Older participants	114,649/345,923 (33.14)
Younger participants	90,981/170,854 (53.25)
Participants with cardiovascular disease	48,552/124,409 (39.02)
Participants without cardiovascular disease	157,078/392,368 (40.03)

a DBP: diastolic blood pressure.

b SBP: systolic blood pressure.

### Changes in SBP Control

**Figure 3.**
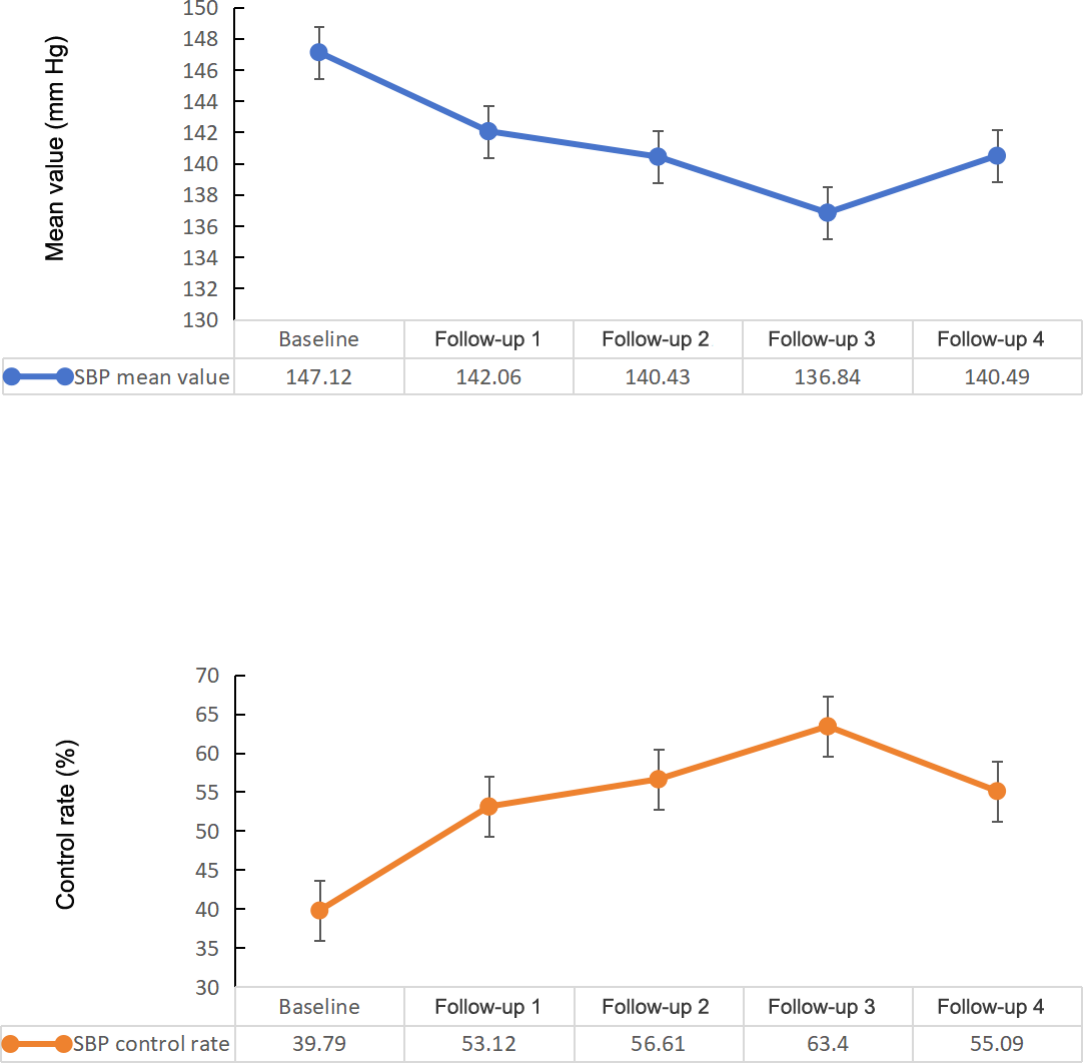
Changes in SBP control. SBP: systolic blood pressure.

The changes in SBP control are described in Figure 3. The SBP values had a linear change trend with continuous time variation (β coefficient=−1.85, 95% CI −1.86 to −1.93; *P*<.001).

The SBP values showed a decreasing trend (Table 2, Figure 3). Compared to baseline, SBP decreased in each quarter by 5.06 mm Hg, 6.69 mm Hg, 10.60 mm Hg, and 6.63 mm Hg (Table 3).

**Table 2.** Slopes of the blood pressure changes from baseline to the fourth follow-up.

Variable	β coefficient (95% CI)		*P* value
SBP^a^	−1.85 (−1.86 to −1.93)		<.001
SBP control rate	0.185 (0.183-0.187)		<.001
DBP^b^	−0.77 (−0.78 to −0.76)		<.001
DBP control rate	0.169 (0.166-0.171)		<.001

a SBP: systolic blood pressure.

b DBP: diastolic blood pressure.

**Table 3.** Degree of change in systolic blood pressure (SBP) control.

Appointment	SBP		SBP control rate	
	β coefficient (95% CI)	*P* value	β coefficient (95% CI)	*P* value
Follow-up 1	−5.00 (−5.11 to −5.00)	<.001	0.18 (0.183-0.187)	<.001
Follow-up 2	−6.69 (−6.74 to −6.63)	<.001	0.60 (0.59-0.61)	<.001
Follow-up 3	−10.60 (−10.54 to −10.63)	<.001	0.76 (0.75-0.77)	<.001
Follow-up ‌4	−6.63 (−6.68 to −6.57)	<.001	1.08 (1.07-1.09)	<.001

SBP control rates for the NEPHSP participants showed an increasing trend (Figure 3, Table 2). Compared to baseline, SBP control rates increased in each quarter to 53.12% (274,493/516,777), 56.61% (292,537/516,777), 63.4% (327,648/516,777), and 55.09% (284,711/516,777; Table 3).

### Changes in DBP Control

**Figure 4.**
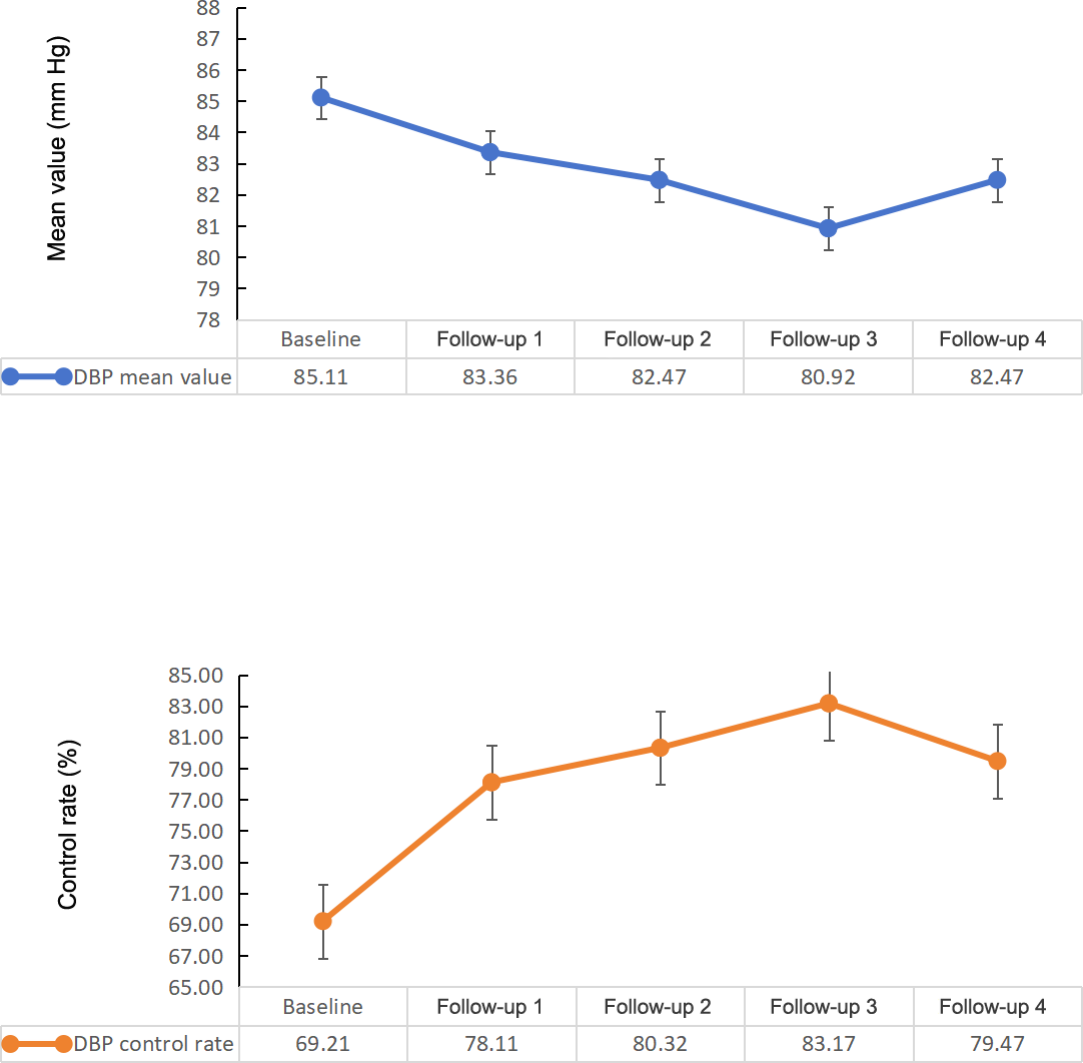
Changes in DBP control. DBP: diastolic blood pressure.

The changes in DBP control are described in Figure 4. The DBP values had a linear trend with continuous time variation (β coefficient=−1.85, 95% CI −1.86 to −1.93; *P*<.001). The DBP values of the NEPHSP participants showed a decreasing trend (Table 2, Figure 4). Compared to baseline DBP, DBP decreased in each quarter by 1.75 mm Hg, 2.64 mm Hg, 4.20 mm Hg, and 2.64 mm Hg (Table 4).

**Table 4.** Degree of change in diastolic blood pressure (DBP) control.

Appointment	DBP		DBP control rate	
	β coefficient (95% CI)	*P* value	β coefficient (95% CI)	*P* value
Follow-up 1	−1.75 (−1.79 to −1.72)	<.001	0.62 (0.61-0.63)	<.001
Follow-up 2	−2.64 (−2.68 to −2.61)	<.001	0.67 (0.66-0.68)	<.001
Follow-up 3	−4.20 (−4.63 to −4.16)	.01	0.89 (0.88-0.90)	<.001
Follow-up‌ 4	−2.64 (−2.68 to −2.61)	<.001	0.61 (0.60-0.62)	<.001

DBP control rates for the NEPHSP participants showed an increasing trend (Table 2, Figure 4). Compared to baseline, DBP control rates increased in each quarter to 78.11% (403,641/516,777), 80.32% (415,062/516,777), 83.17% (429,829/516,777), and 79.47% (410,662/516,777; Table 4).

### Sensitivity and Stratified Analyses

Our sensitivity analysis stratified by the predefined subgroups showed that a decrease in blood pressure and an increase in blood pressure control rates were consistent across subgroups of age, sex, and a diagnosis of baseline CVD. The parameters of the sensitivity analysis are accessible in Multimedia Appendix 3. The changes in SBP were converse between the baseline controlled SBP and uncontrolled SBP groups. The baseline controlled SBP group had an increased control of their SBP, while the baseline uncontrolled SBP group had a decreased control of their SBP. The SBP control rates decreased in the baseline controlled SBP group, while the SBP control rates increased in the baseline uncontrolled SBP group.

**Figure 5.**
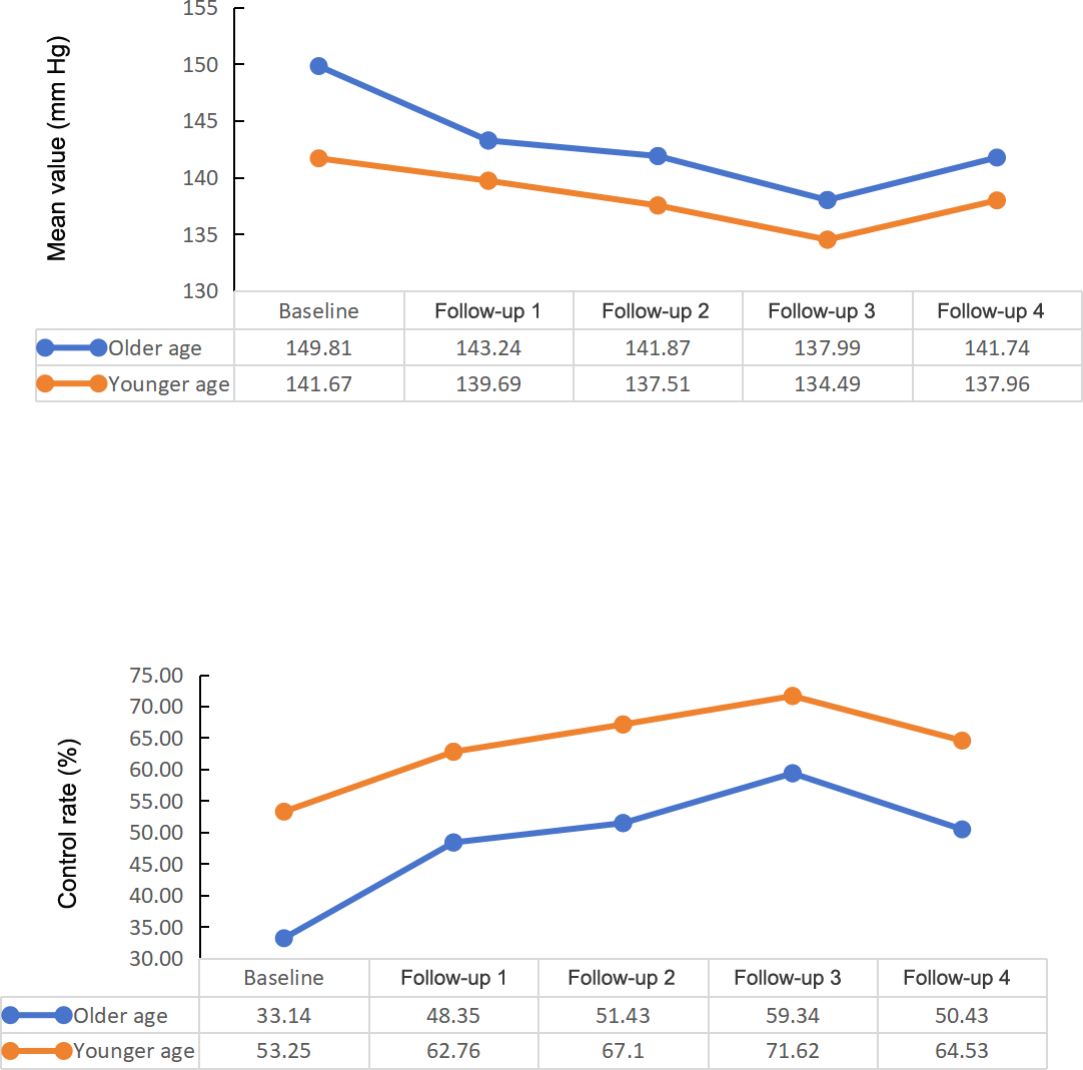
Systolic blood pressure control of the age groups.

The changes in SBP control in the older and younger age groups are described in Figure 5. Compared with younger age group, the older age group showed a larger decrease in SBP and a larger increase in SBP control rates. The SBP of the older age group were higher than the younger age group. The SBP control rates of the older age group were significantly lower than the younger age group (*P*<.001).

**Figure 6.**
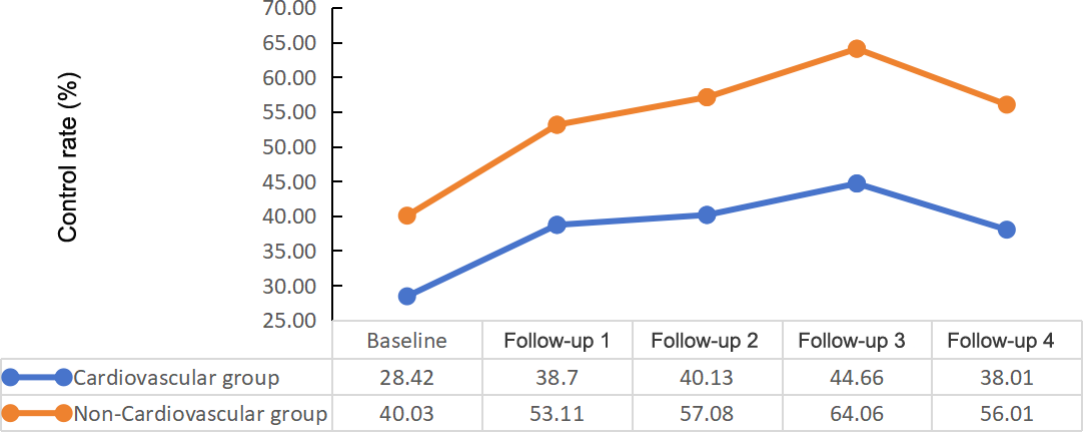
Systolic blood pressure control of the cardiovascular disease group.

The changes in SBP control of the participants with and without CVD were described in Figure 6. The participants with CVD had a smaller decrease in SBP and smaller increase in SBP control rates than the participants without CVD. The SBP control rates of the participants with CVD were significantly lower than the participants without CVD (*P*<.001).

The stratified analysis by sex demonstrated that the decrease in SBP values and the increase in SBP control rates were greater in the female group than in male group; however, the differences were relatively small.

## Discussion

### Principal Findings

To our knowledge, this is the first study that longitudinally explored effect of the NEPHSP on blood pressure control. The study findings demonstrated that the NEPHSP was effective for improving SBP and DBP control. The NEPHSP decreased the values of SBP and DBP and increased the control rates of SBP and DBP.

The routine follow-up that monitored blood pressure and the individualized interventions of the NEPHSP might contribute to the improvement of SBP and DBP control. The routine follow-ups generated clinically crucial data that could recognize prioritization of the participant subgroups who had poorly controlled blood pressures [35]. Previous studies also reported that the regular population review that enables the formulation of customized interventions for improving blood pressure control is an effective strategy for hypertension management [35-48]. Individualized interventions were formulated according to the evaluations of routine follow-ups to identify appropriate strategies to improve blood pressure control in participants with poorly controlled blood pressure [35,36,47].

Although the NEPHSP improved the control of blood pressure, the mean values of SBP were still slightly higher than the normal criteria. This might be caused by therapeutic inertia [33]. Therapeutic inertia was defined as a failure of health care professionals to initiate or intensify treatment appropriately during visits [33,49]. Previous research demonstrated that the failure to appropriately initiate or intensify treatment was introduced by the perspective of health care professionals that near-target blood pressures were acceptable and the overconcern about adverse effects caused by treatment intensification [47,48,50,51]. Because of the doctor-related factors leading to therapeutic inertia, participants with near-target blood pressures might not accept intensification of antihypertensive treatments, which could contribute to the slightly higher values of blood pressure [47,48,50,51]. Although therapeutic inertia is associated with poor blood pressure control and cardiovascular events, therapeutic inertia is common in hypertension management in primary care settings [47,48,51]. Given this situation, interventions for handling therapeutic inertia are important for improving hypertension management in primary care settings [33,47,48,51]. However, in terms of the NEPHSP, no component for tackling therapeutic inertia were embedded. The relevant components addressing therapeutic inertia can be considered for inclusion in the NEPHSP [33].

The subgroup analysis showed that the older participants had greater blood pressure improvement compared to the younger participants. This finding might be relevant to the referral mechanism of the NEPHSP. Compared with younger patients with hypertension, older patients with hypertension have more complex health conditions, such as frailty, comorbidities, and polypharmacy [34,52-58]. These complex health conditions can introduce challenges for blood pressure control in primary care settings, which are positively associated with poor blood pressure control [34,52-58]. To improve poor blood pressure control in older patients with complex health conditions in primary care settings, a referral mechanism to superior medical institutions is important [34,46,47,57-59]. In terms of the NEPHSP, the older participants with complex health conditions and poor blood pressure control were recognized via the regular follow-up evaluations. Based on the health condition evaluations of the regular follow-ups, the older participants could accept more referrals to superior medical institutions than the younger participants. Through the referral to superior medical institutions, the older participants could receive more intensive antihypertensive treatments than the treatments in primary care settings [34,46,47,57-59]. Because of the intensive antihypertensive treatments provided in superior medical institutions, the older participants had more blood pressure control improvement than the younger participants [34,46,47,57-59].

Although older participants had a greater improvement in blood pressure control than younger participants, the SBP of the older participants were still higher than the younger participants. Moreover, the SBP control rates for the older participants were significantly lower than the younger participants. These study findings might be associated with concerns about adverse events caused by blood pressure–lowering pharmacotherapy [33,34,45-58]. However, previous robust research evidence demonstrated that decreasing the blood pressures of older adults to normal range is safe and effective for reducing risks of cardiovascular events [59,60]. Consequently, older people with hypertension should have a similar attitude toward blood pressure control as younger people with hypertension [59,60]. Given this situation, strategies to intensify blood pressure control in older participants engaging in the NEPHSP are necessary [59,60].

The improvement in the blood pressure control for the participants with CVD was less than the participants without CVD, while blood pressure control for people with hypertension and CVD needs to be more intensive than people with hypertension but without CVD [60]. Similar to the older participants, the patients with hypertension with CVD also required more referrals to superior medical institutions to achieve optimal blood pressure control [61-66]. Compared to patients with hypertension without CVD, the patients with CVD demand more intensive blood pressure control to prevent cardiovascular events, and blood pressure control in patients with hypertension and CVD is challenging in primary care settings [61-66]. The main challenges are potential adverse drug effects caused by polypharmacy and emergent cardiovascular events caused by intensive antihypertensive treatments [61-66]. Given this situation, referral to superior medical institutions is necessary for patients with hypertension and CVD to achieve optimal blood pressure control via intensive and safe antihypertensive treatments [61-66].

Although the NEPHSP had the algorithm to evaluate health conditions of the participants with comorbidities and subsequent referral mechanism to superior medical settings, the improvement of blood pressure control in the participants with CVD was still less than the participants without CVD. The lesser improvement in blood pressure control in the participants with CVD could be caused by the health evaluations of the regular follow-ups. The health condition evaluations might fail to accurately assess the health conditions of the participants with CVD, as the evaluations did not have systematic tools such as checklists, which could lead to failure to refer the participants who needed referral [61-66]. Furthermore, no explicit criteria for referral of participants with CVD were predefined, which could lead to confusion for the primary health care professionals when making the decisions on referral [61-66]. Given that the blood pressure control in people with hypertension and CVD needs to be more intensive than people without CVD, strategies for intensifying blood pressure control in the NEPHSP participants with CVD should be considered [61-66].

The subgroup analysis showed that the participants with controlled SBP at baseline had an increase in SBP and a decrease in SBP control rates, which were converse to the SBP results of all study participants. These results could be associated with the health evaluation algorithm of the regular follow-ups of the NEPHSP. According to the health evaluation algorithm, the participants who had controlled blood pressures that were evaluated in the regular follow-ups would not accept specific interventions. Because specific interventions were not provided, the participants with controlled SBP at baseline could have an increase in SBP and a decrease in SBP control rates. Although SBP of the participants was controlled at baseline, the increase of SBP values within the normal range could also increase CVD events [58]. Given this situation, the health evaluation algorithm and interventions of the NEPHSP for the participants with controlled SBP at baseline were suggested to be reformulated [58-61].

Blood lipid and glucose control could impact hypertension management. Previous studies reported that the triglyceride-glucose index (TyG), which is calculated by multiplying fasting triglyceride by fasting glucose, had a positive relationship with blood pressure control [31,32,61,62]. The increase of the TyG could contribute to poor blood pressures control [31,32,61,63]. A linear dose-response relationship between changes in the TyG and the change in blood pressure was identified [31,32,62,63]. To achieve optimal blood pressure control, blood lipid and glucose should be concurrently managed in people with hypertension [31,32,62,63]. However, in terms of the NEPHSP, no treatment targets for blood lipid and glucose were predefined for the study participants, which might cause ignorance toward blood lipid and glucose control. The ignorance of blood lipid and glucose control might cause a high TyG, which could contribute to the near-target blood pressure levels in this study [31,32,62,63]. Given this situation, further studies are required to confirm whether blood lipid and glucose control impact blood pressure control of the NEPHSP participants, in order to clarify the target for improving blood pressure control in this population.

### Limitations

This study had several limitations. Although the effects of the NEPHSP on blood pressure control were modest, this study could not confirm the factors impacting blood pressure control of the NEPHSP. The group divisions for subgroup analysis were general and not explicit. Further studies that include a gradient group division to explore factors impacting hypertension control of NEPHSP are needed. Randomization for group division was not applied in the subgroup analysis, which could lead to an uneven distribution of confounding factors. Further studies that use randomization or matched group division are need to confirm the factors impacting hypertension control of the NEPHSP. This study explored baseline characteristics that could impact hypertension control of the NEPHSP, and future studies exploring the impact of the factor trajectories on hypertension control are needed. In addition, this study evaluated NEPHSP in a pilot city, and further nationwide studies are needed.

The blood pressure data in this study were the clinical records obtained from routine blood pressure measurements that were conducted via the Riva-Rocci and Korotkoff techniques [64-66]. The Riva-Rocci and Korotkoff techniques are still considered a cornerstone in evaluation of blood pressure levels in clinical trials [64-66]. However, the techniques have limitations, including that they can provide only a limited number of blood pressure values and can not reflect blood pressure fluctuations over 24 hours [64-66]. As a result, potential influences of blood pressure fluctuations over 24 hours in our study findings were uncertain [64-66]. Further studies that use advanced devices such as ambulatory blood pressure monitoring to explore impacts of 24-hour blood pressure fluctuations on effects of the NEPHSP on blood pressure control are required. Despite the uncertainty of impacts of 24-hour blood pressure fluctuations, our study findings also provide preliminary evidence for the control of blood pressure by the NEPHSP. First, our study was conducted at population level. The large scale of the study participants might reduce the impact of 24-hour blood pressure fluctuations on the changes in blood pressure levels to some degree. Second, the follow-up time was one year for this study, which could offset the impact of blood pressure fluctuations over 24 hours.

This study was designed as a self-controlled longitudinal study. We compared blood pressure levels before the participants accepted health services from the NEPHSP versus after accepting the health services. The changes in blood pressure levels could preliminarily reflect the effect of the NEPHSP on blood pressure control for the participants with hypertension. However, the data monitoring blood pressure changes were only available for the NEPHSP participants and were inaccessible for people with hypertension who did not register with the NEPHSP. Because of the inaccessibility of blood pressure monitoring data for this population, this study was unable to compare blood pressure control between the NEPHSP participants and people who did not register with the NEPHSP. Given this situation, further cohort and control trials are required to confirm advantages of the NEPHSP.

### Conclusion

The NEPHSP was effective for improving blood pressure control of Chinese people with hypertension. However, the SBP of the study participants was near-target, which could imply therapy inertia in the NEPHSP participants. A suggested strategy to tackle potential therapy inertia was to intensify blood pressure control of the NEPHSP. The effects of the NEPHSP on blood pressure control in older participants and participants with CVD were modest. Embedding intensive treatment modules into the NEPHSP was suggested to improve blood pressure control of older participants and participants with CVD. Further studies confirming effects of the NEPHSP on blood pressure control for people with specific characteristics, such as older age and comorbidities, are required.

## Supplementary material

10.2196/65783 Multimedia Appendix 1 Univariate regression analysis of covariates.

10.2196/65783 Multimedia Appendix 2 Corresponding information of each covariate in the generalized estimating equation regressions.

10.2196/65783 Multimedia Appendix 3 Subgroup analysis.

## Abbreviations

CVD: cardiovascular disease
DBP: diastolic blood pressure
GEE: generalized estimating equation
NEPHSP: National Essential Public Health Service Package
SBP: systolic blood pressure
TyG: triglyceride-glucose index
